# Are Epiphytic Microbial Communities in the Carposphere of Ripening Grape Clusters (*Vitis vinifera* L.) Different between Conventional, Organic, and Biodynamic Grapes?

**DOI:** 10.1371/journal.pone.0160852

**Published:** 2016-08-08

**Authors:** Elizabeth Kecskeméti, Beate Berkelmann-Löhnertz, Annette Reineke

**Affiliations:** Department of Phytomedicine, Geisenheim University, Geisenheim, Germany; University of California Davis, UNITED STATES

## Abstract

Using barcoded pyrosequencing fungal and bacterial communities associated with grape berry clusters (*Vitis vinifera* L.) obtained from conventional, organic and biodynamic vineyard plots were investigated in two subsequent years at different stages during berry ripening. The four most abundant operational taxonomic units (OTUs) based on fungal ITS data were *Botrytis cinerea*, *Cladosporium* spp., *Aureobasidium pullulans* and *Alternaria alternata* which represented 57% and 47% of the total reads in 2010 and 2011, respectively. Members of the genera *Sphingomonas*, *Gluconobacter*, *Pseudomonas*, *Erwinia*, and *Massilia* constituted 67% of the total number of bacterial 16S DNA reads in 2010 samples and 78% in 2011 samples. Viticultural management system had no significant effect on abundance of fungi or bacteria in both years and at all three sampling dates. Exceptions were *A*. *alternata* and *Pseudomonas* spp. which were more abundant in the carposphere of conventional compared to biodynamic berries, as well as *Sphingomonas* spp. which was significantly less abundant on conventional compared to organic berries at an early ripening stage in 2011. In general, there were no significant differences in fungal and bacterial diversity indices or richness evident between management systems. No distinct fungal or bacterial communities were associated with the different maturation stages or management systems, respectively. An exception was the last stage of berry maturation in 2011, where the Simpson diversity index was significantly higher for fungal communities on biodynamic compared to conventional grapes. Our study highlights the existence of complex and dynamic microbial communities in the grape cluster carposphere including both phytopathogenic and potentially antagonistic microorganisms that can have a significant impact on grape production. Such knowledge is particularly relevant for development, selection and application of effective control measures against economically important pathogens present in the grape carposphere.

## Introduction

Grapevines, cultured on approximately 7.5 million ha in vineyards throughout temperate regions worldwide [1], are hosts for complex communities of microorganisms and arthropods, which can significantly influence both the quantity of the yield as well as the quality of must and wine [2, 3]. Some of the organisms present in the grape carposphere (fruit surface) or phyllosphere (leaf surface) can act as pathogens, in particular fungi as well as bacteria or viruses [4, 5]. Others serve as mutualists, including mycorrhizal fungi, plant growth promoting bacteria colonizing roots [6], and endophytes inside grapevine leaves or stems [7]. Associated microorganisms present inside or on grape berries are also known to influence flavour of grapes and wines by producing volatile organic compounds [8].

To prevent detrimental economic levels of injury inflicted by pathogenic organisms to grapes, different pest management programs and viticultural farming/cultivation systems are applied by the growers. In conventional viticulture growers follow the conceptual framework of integrated pest management (IPM), which includes careful monitoring, preventive cultural practices and, once economic threshold levels are reached, a combination of the use of synthetic pesticides and biological control strategies. Alternatively, growers follow the principles of organic or biodynamic winegrowing, respectively. In organic viticulture, grapes are typically cultivated without the use of chemical fertilizers or synthetic pesticides. Instead, growers encourage biological control and use crop protectants and fertilizers from naturally produced sources, such as botanical pesticides or animal byproducts [9]. Pest and disease management systems in organic viticulture also include the application of copper, especially for the control of downy mildew (*Plasmopara viticola*) or sulphur against powdery mildew (*Erysiphe necator*). In biodynamic viticulture application of artificial chemical products is not permitted, and specific biodynamic preparations made from cow manure, silica and plant extracts are used as soil stimulants and plant growth/health promoters [10]. Similar to organic viticulture, copper and sulphur applications against grape pathogens are allowed in biodynamic viticulture.

The ascomycete *Botrytis cinerea* Pers.: Fr. (teleomorph *Botryotinia fuckeliana* (de Bary) Whetzel) is regarded as one of the most destructive fungi in cool climate viticulture, causing *Botrytis* bunch rot or grey mould of grapes and changing the physiochemical state of grape berries dramatically [11]. Several other microorganisms are involved in the development of bunch rot, including fungi from the genera *Penicillium*, *Trichothecium*, *Aspergillus*, *Alternaria*, *Cladosporium*, and *Rhizopus*, as well as bacteria from the genera *Acetobacter* and *Gluconobacter* [12–14]. Currently, application of synthetic *Botrytis* fungicides in combination with management practices like grape cluster division or leaf removal influencing microclimatic conditions in the fruit zone are effective methods to reduce *Botrytis* bunch rot severity [15, 16]. Recently, extreme weather conditions in central Europe such as heavy rainfall events during the ripening period have coincided with earlier maturation of grape berries. Together, these lead to an increases in berry bunch rot, which is increasingly difficult to control. This frequently results in substantial crop losses and negatively impacts must quality [14]. Repeated use of fungicides could induce resistance in target organisms [17–19] and impact the natural yeast flora, leading to changes in wine quality if yeasts are not added during the fermentation process [3, 20]. As an alternative to fungicide applications the use of biological control agents (BCAs) offer a tool to suppress *B*. *cinerea* and other bunch rot organisms on grapes [21]. The most commonly used BCAs in viticulture are fungi of the genera *Trichoderma*, *Aureobasidium*, and *Sporidiobolus* [22–24], bacteria like *Bacillus* spp. or *Pseudomonas* spp. [25], as well as yeasts from the genera *Metschnikowia*, *Pichia* or *Candida* [26–28]. Since these BCAs are often part of the natural microbiota of grape leaves and berries, knowledge about the structural and functional diversity of the microbial community present on grape berry skins is a key to promoting indigenous antagonists by certain viticultural practices or management systems.

In the present study we analysed the epiphytic fungal and bacterial community structures in the carposphere and rachis of grape berries obtained from vineyard plots managed according to standards of conventional (integrated), organic and biodynamic viticulture. The respective viticultural systems were sampled in two subsequent years and at different stages during berry ripening for analysis using barcoded pyrosequencing. In particular, the following questions were investigated: Are there associations between the viticultural management system and the composition of fungal and bacterial communities in the grape carposphere? Does the microbiocoenosis of the grape cluster carposphere vary between different stages of grape maturation? Is a vintage effect evident, or, in other words do we see an influence of the climatic conditions of the respective growing season on the composition of microbial communities in the grape carposphere? And finally, is the occurrence and quantity of putative *Botrytis* antagonists influenced by the management system?

## Material and Methods

### Study site and sampling of grapes

Grape clusters were sampled in one vineyard owned by Geisenheim University and located in Geisenheim in the German grapevine growing region Rheingau (49°59’N, 7°56’E). This vineyard contained plots where three different management systems (conventional, organic and biodynamic) have been applied side by side since 2006 (S1 Fig). The experimental design was a complete block design, where each management system was represented by four blocks, each consisting of four rows with 32 grape plants per row (S1 Fig). The vines (*Vitis vinifera* L. cv. Riesling; clone Gm 198–30; rootstock either SO4 or Börner) were planted in 1991 in an area of 0.8 ha with a growing space of 2.4 m^2^ (root distance 1.2 m and line spacing 2 m). The vineyard plots were managed according to (i) the general principles of integrated pest management (IPM) in viticulture as listed in Annex III of the Directive 2009/128/EC [29] for the conventional plots, (ii) standards of the European Council Regulation (EC) No 834/2007 [30] and ECOVIN for the organic plots and (iii) EC No 834/2007 and DEMETER for the biodynamic plots. In the conventional plots, eleven applications of fungicides were performed following a common scheme applied by growers producing grapes according to the rules of IPM. Products and active ingredients applied between mid-April to mid-August during the years 2006–2011 are listed in S1 Table. In the organic and biodynamic plots, nine to eleven applications of plant protection products allowed in these management systems occurred (S1 Table), including copper and sulphur based products as well as plant resistance inducers. In addition, biodynamic preparations based on compost or cow dung were applied in the biodynamic plots. Insecticides were not applied in either of these two viticultural systems. In all plots, cover crops were planted in alternating alleys as described in Döring et al. [31]. Cover crops in conventional plots consisted of a perennial grass mix, while cover crops in the organic and biodynamic plots were composed of a mixture of herbs, grasses and legumes, including perennial broad-leaved flowering plants. For a detailed description of the experimental site see Döring et al. [31].

Between end of August and October 2010 and 2011, respectively, eight grape clusters were collected separately in each plot (two grape clusters per replicate, see S1 Fig) at three different stages (according to the BBCH scale (Biologische Bundesanstalt, Bundessortenamt und Chemische Industrie) during the ripening period of berries: (i) beginning of ripening (stage BBCH 81); (ii) softening of berries (stage BBCH 85); (iii) berries ripe for harvest (stage BBCH 89). Grape plants used for sampling were marked in order to assure that sampling was always done in the same eight plants in both consecutive years. In total, 144 grape clusters were sampled and were used for DNA extraction of microorganisms.

### Isolation of microorganisms from the grape carposphere

Microorganisms from the carpospheres and rachis of the 144 sampled grape clusters were isolated as described by Laforgue et al. [32] with slight changes. Each grape cluster was put into a 200 mL centrifuge flask and was covered with approximately 50 mL sterile washing solution (distilled water supplemented with 0.2% Tween 80). The flasks were sonicated for 3 min and were shaken overhead for 30 min. The grape clusters were removed and the liquid was centrifuged at 8000 rpm, 30 min and 22°C. The obtained pellets were frozen at -20°C until DNA extraction.

### DNA extraction, ITS amplification and pyrosequencing

From each pellet obtained from grape clusters, total genomic DNA was extracted using the PowerSoil DNA Isolation Kit (Süd-Laborbedarf, Gauting, Germany) according to the manufacturer’s instructions. For lysis and homogenization Precellys 24 (Peqlab Biotechnology, Erlangen, Germany) was used. DNA qualities were checked electrophoretically on a 1% agarose gel in 1% Tris-acetate-EDTA (TAE) buffer, stained with SYBR-Safe (Invitrogen, Karlsruhe, Germany). DNA concentrations were measured using a NanoDrop 1000 Spectrophotometer (Peqlab Biotechnology, Erlangen, Germany).

For amplification of conserved fungal and bacterial genes, DNAs from two samples per year, management system and date were pooled together in equimolar amounts, resulting in a total of 72 pooled DNA samples for pyrosequencing.

The variable region of the fungal ITS was amplified with the primer pair ITS1(5’-CTT GGT CAT TTA GAG GAA GTA A-3’) and ITS2 (5’-GCT GCG TTC TTC ATC GAT GC-3’) [33]. Additionally, the V1-V2 region of the bacterial 16S rDNA gene was amplified with the primer pair 27F (5’-MGA GTT TGA TCC TGG CTC AG-3’) and 337R (5’-GCT GCC TCC CGT AGG AGT-3’) [34]. Primers were modified by the addition of a GS FLX Titanium Key-Primer A and B (A: CGT ATC GCC TCC CTC GCG CCA and B: CTA TGC GCC TTG CCA GCC CGC), a four-base library “key” sequence (TCAG) and a multiplex identifier (MID) sequence.

The PCR reaction was set up in a total volume of 30 μL containing 20 ng of pooled DNA samples, 10 pmol of each primer, 10 mM dNTP, 10x DreamTaq Buffer and 1 U of the high fidelity DreamTaq DNA polymerase (Fermentas, St. Leon-Rot, Germany) and was performed with an initial denaturation step at 94°C for 4 min, followed by 30 cycles of denaturation at 94°C for 30 s, annealing at 55°C for 1 min, extension at 72°C for 90 s and a final extension at 72°C for 10 min. An aliquot of each PCR product (~400 bp) was checked for successful amplification on a 1% agarose gel in 1% TAE buffer, stained with SYBR-Safe (Invitrogen, Karlsruhe, Germany). Remaining PCR products were purified with HiYield PCR Clean-up/Gel Extraction Kit (Süd-Laborbedarf, Gauting, Germany) and DNA concentrations were measured using NanoDrop 1000 Spectrophotometer (Peqlab Biotechnology, Erlangen, Germany). Tag-encoded fungal and bacterial PCR products were pooled in equimolar concentrations and 454 pyrosequencing was carried out commercially on a Roche (454) FLX Genome Sequencer (LGC Genomics GmbH, Berlin, Germany).

### Operational taxonomic unit-based (OTU) sequence analysis

Processing, quality filtering and clustering of 454 reads into operational taxonomic units (OTUs) was performed using the automated pipeline CloVR-ITS for the characterization of fungal communities [35] and CloVR-16S for the characterization of bacterial biota [36], respectively. The obtained OTUs defined at species (3% sequence dissimilarity) and genus level (5% sequence dissimilarity), respectively, were used to calculate fungal and bacterial diversity indices (Shannon index and Simpson index), richness estimator (Chao1) and rarefaction curves using software PAST [37], which has been used for similar purposes before [38]. One-way ANOSIM (analysis of similarities based on OTUs without singletons) implemented in PAST was used to evaluate the effects on fungal and bacterial community structure between the different management systems, sampling dates and years [37]. ANOSIM creates a test statistic of R, which indicates if differences between samples exist. Interpretation of R values was according to Clarke [39] with the following categories: separated R > 0.75, clearly different R > 0.5, and barely separable R < 0.25. Prior to ANOSIM, non-metric multidimensional scaling (NMDS) of the samples was done based on the Bray-Curtis dissimilarity matrix using an algorithm implemented in PAST [37] after Bonferroni correction. Principal component analysis (PCA) in PAST was used to assess relationships in fungal and bacterial communities, respectively, along the three different sampling time points.

Further data analyses were performed using STATISTICA software package, version 7.1 (StatSoft, Tulsa, Oklahoma, USA). Data of both the four most frequent fungal and five most frequent bacterial OTUs as well as diversity indices and richness estimator were tested for normal distribution (Shapiro-Wilk test; α = 0.05). Respectively, a one-way ANOVA or a non-parametric Kruskal-Wallis-ANOVA was performed with normally and not normally distributed data, followed by Tukey HSD test (α = 0.05) or multiple comparisons using Kruskal-Wallis test (α = 0.05). Data of microbial community as well as diversity and richness indices between the sampling years were analyzed for normal distribution (Kolmogorov-Smirnov test; α = 0.05) and for homogeneity of variance (Levene’s test; α = 0.05). A t-test (α = 0.05) or a Mann-Whitney U test (α = 0.05) was performed with normally and not normally distributed data, respectively.

### Quantitative PCR analysis of potential fungal antagonists

Presence of the two putative fungal antagonists *Aureobasidium pullulans* and *Sporidiobolus pararoseus* was further assessed using qPCR in single grape berry samples obtained at the beginning of ripening (BBCH 81) in 2010 in all three plots. Primer pair ApuIIF1 (5’-GAT CAT TAA AGA GTA AGG GTG CTC A-3’) and ApuIIR1 (5’-GCT CGC CTG GGA CGA ATC-3’) [24] was used to quantify *A*. *pullulans* ITS copies. Primer pair SporiF1 (5’-GAT CTC TTG GCT CTC GCA TC-3’) and SporiR1 (5’-acg ctc aga atc caa cac c-3’) (personal communication Florian Schmid) was used to quantify *S*. *pararoseus* ITS copies. Primer pair Bb-actin-f (5’-AAG TCC AAC CGT GAG AAG ATG AC-3’) and Bb-actin-r (5’-ATC ACC AGA GTC GAG GAC GAT AC-3’) for actin (GenBank accession no. GT897142) and primer pair ITS1f (5’-TCC GTA GGT GAA CCT GCG G-3’) and ITS1r (5’-GCT GCG TTC TTA TCG ATG C-3’) for ITS [40] were used as reference genes for quantification of total fungal actin and ITS copy numbers. Primers were checked for specificity prior to qPCRs. All qPCR reactions were performed in three technical replicates and set up in a total volume of 25 μL containing 2 μL of 1:10 diluted target DNA, 10 pmol of each species specific forward and reverse primer and 12.5 μL F-416 2X DyNAmo Color-Flash Master Mix (Finnzymes, Espoo, Finnland). Water served as a no-template control. Real-time PCR was performed in a two-step cycling program using an iCycler iQ5 PCR machine (BioRad Laboratories, Hercules, USA) with an initial denaturation step at 95°C for 3 min, followed by 40 cycles of denaturation at 95°C for 10 s and annealing at 60°C for 30 s.

Quantification cycle (C_q_) values were calculated using the iQ5 version 2 software (BioRad, Munich, Germany). Relative DNA amounts of the target species present in the respective samples were normalized based on three independent technical replicates with relation to mean C_q_ values of the two fungal reference genes. Quantification of relative DNA amounts were calculated using the method implemented in qBase software [41], which allows the inclusion of multiple reference genes for normalization and corrects for different amplification efficiencies. Statistical differences in the relative amounts of *A*. *pullulans* and *S*. *pararoseus* DNAs between the samples were calculated using STATISTICA software package, version 7.1 (StatSoft, Tulsa, Oklahoma, USA). Data were tested for normal distribution (Shapiro-Wilk test; α = 0.05). A non-parametric Kruskal-Wallis-ANOVA was performed with not normally distributed data, followed by multiple comparisons using Kruskal-Wallis test (α = 0.05).

### Climate data

Data for air temperature and precipitation were obtained from a weather station (station “Geisenheim”, 49°59’N, 7°56’E) of the German meteorological service DWD (Deutscher Wetterdienst) located next to the experimental vineyard in Geisenheim and are shown in S2 Fig.

## Results

The purpose of the present study was to assess fungal and bacterial communities present on the carposphere and rachis of grape berries sampled in different vineyard management systems and during different stages of berry maturation. For this purpose, 144 DNA grape samples were submitted to pyrosequencing of fungal ITS and bacterial 16S sequences, respectively.

### Fungal communities in the grape carposphere

A total number of 98,950 fungal ITS sequences (including 1,602 singletons) passed the quality control. The total number of reads obtained per year (without singletons) was 47,013 in grape samples obtained in 2010 and 50,335 in samples collected in 2011, respectively. From the total number of reads in 2010, 17,937 OTUs were identified in the biodynamic plot, while 14,778 OTUs were identified in the organic and 14,298 OTUs in the conventional plot, respectively. In 2011, 19,567 OTUs were identified in the organic plot, 16,576 OTUs in the biodynamic and 14,192 OTUs in the conventional plot, respectively.

Rarefaction curves of all fungal samples obtained from the carposphere of grape clusters in both years reached an asymptote and were close to saturation, indicating that the sampling size was acceptable (S3 Fig).

**Fig 1:**
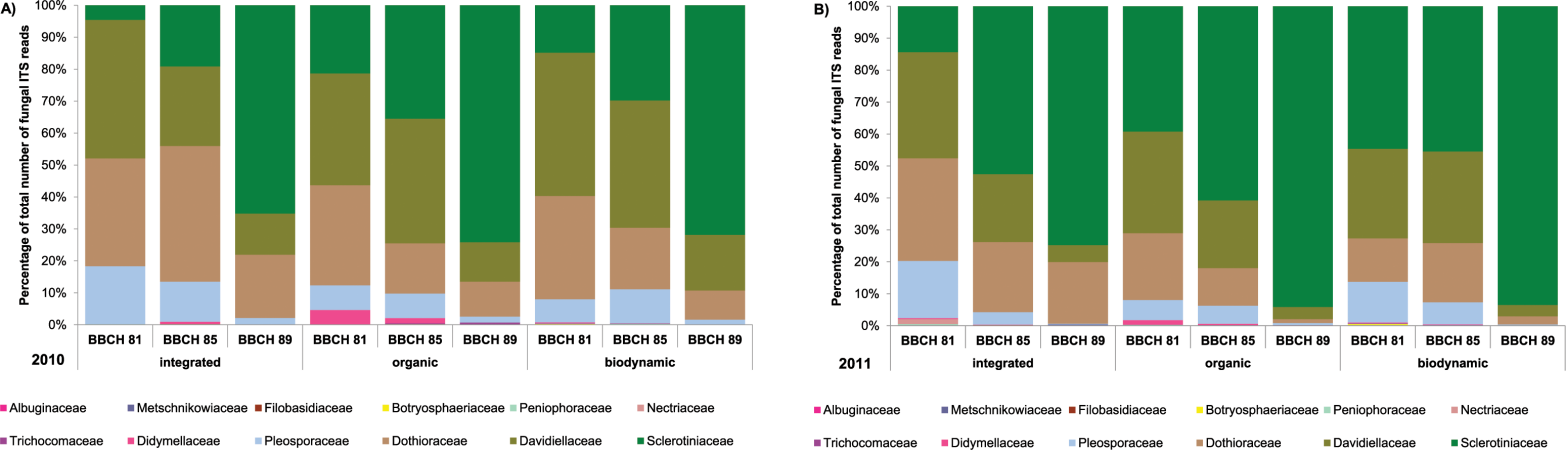
**Taxonomic distribution of fungal communities in the carposphere of conventional, organic and biodynamic grape clusters in 2010 (A) and 2011 (B).** The distribution of the reads indicates the number of OTUs in each fungal family based on ITS sequence data. Grapes were sampled at three stages of berry maturation (BBCH 81, BBCH 85 and BBCH 89). Only fungal families contributing to at least 0.2% of the obtained reads are shown.

**Fig 2:**
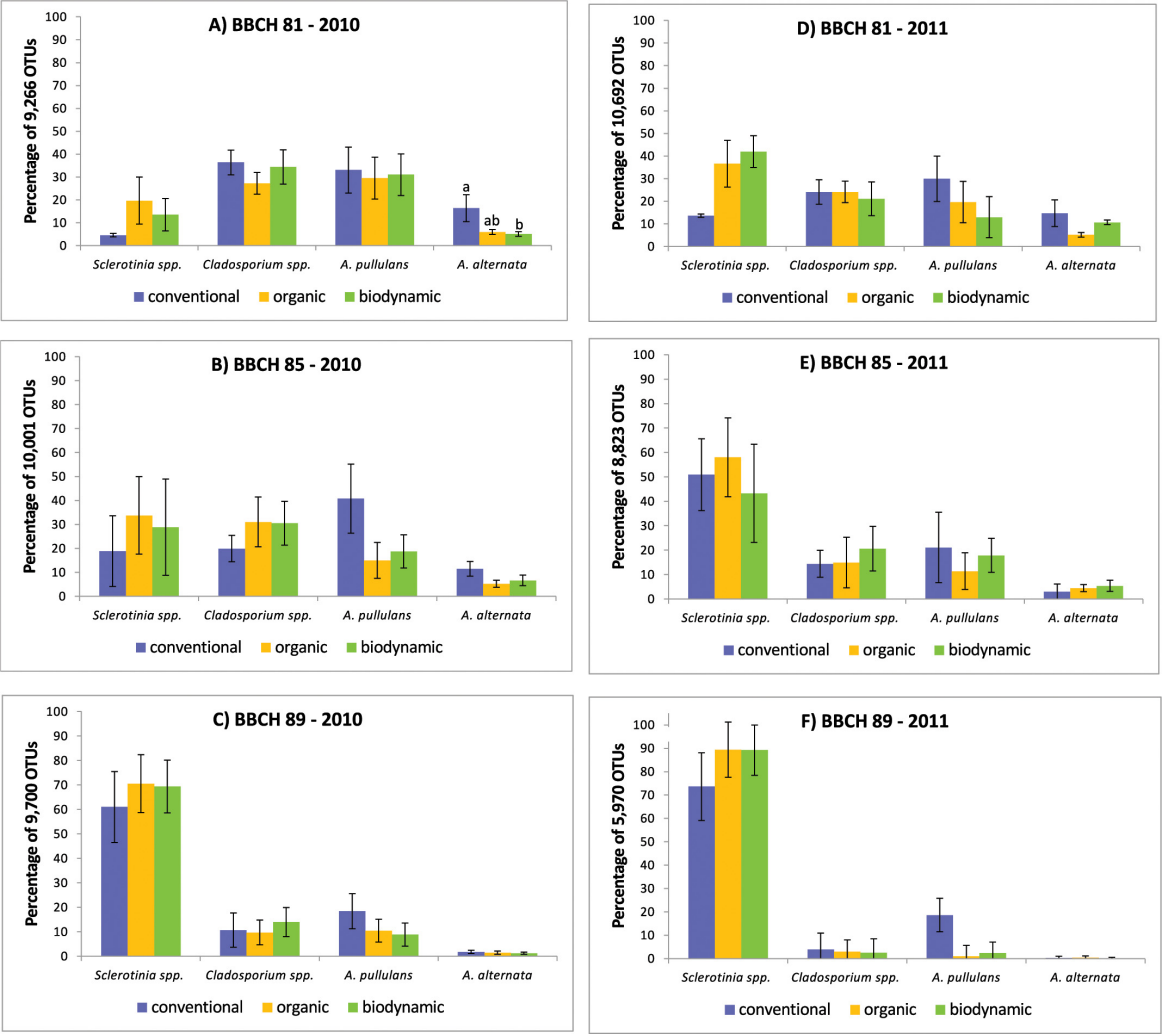
Percentage of identified OTUs belonging to the four most abundant fungal species. Grapes were sampled in conventional (blue), organic (yellow) and biodynamic (green) vineyard plots in 2010 (A-C) and 2011 (D-F), respectively, at three different stages of maturation (A, D: BBCH 81, beginning of ripening; B, E: BBCH 85, softening of berries; C, F: BBCH 89, berries ripe for harvest). Bars indicate standard errors of four pooled grape samples. Significant differences between abundance of individual fungal species and management systems within the same year and stage of berry ripening are indicated by different letters (Kruskal-Wallis test, *P* < 0.05).

To identify the most frequent fungal taxa present in the grape carposphere samples, OTUs were clustered with all obtained reads. Unclassified fungi formed a fairly large proportion of fungal richness (38% of OTUs identified in the 2010 samples and 49% of OTUs in the 2011 samples, respectively). Taxonomic distribution of fungal ITS data based on proportions of OTUs at the family level is shown in Fig 1. At species level, the four most abundant fungal OTUs identified were *Botrytis cinerea*, followed by *Cladosporium* spp., *Aureobasidium pullulans* and *Alternaria alternata* (Fig 2). These four most abundant OTUs represented 57% of the total reads in 2010 (S2 Table) and 47% of the total reads in 2011 (S3 Table).

Obtained OTUs were analyzed regarding statistical differences in the structural composition of fungal microbiota present in the carposphere of berries sampled in plots with different management systems and at different maturation stages. In all samples, the abundance of *B*. *cinerea* increased during the ripening period in both years (Fig 2). Also, the abundance of *B*. *cinerea* was significantly higher at all maturation stages in 2011 than in 2010 (*P* = 0.0469 for BBCH 81; *P* = 0.0433 for BBCH 85; *P* = 0.000 for BBCH 89). In contrast, the abundance of *Cladosporium* spp., *A*. *pullulans* and *A*. *alternata* decreased during the ripening period (Fig 2). Abundance of *Cladosporium* spp., *A*. *pullulans* and *A*. *alternata* was significantly higher in 2011 than in 2010 at BBCH 89 (*P* = 0.000 for *A*. *alternata; P* = 0.0153 for *A*. *pullulans* and *P* = 0.0433 for *Cladosporium* spp.). *A*. *alternata* was also more abundant in grape samples obtained in 2011 than in 2010 (*P* = 0.0407). Viticultural management system had no significant effect on abundance of these four fungi with respect to year or sampling site, with the exception of *A*. *alternata*. In 2010, this fungus was more abundant on berries from the conventional plots than on berries from the biodynamic plots at the beginning of berry ripening (BBCH 81) (*P* = 0.0243) (Fig 2).

**Fig 3:**
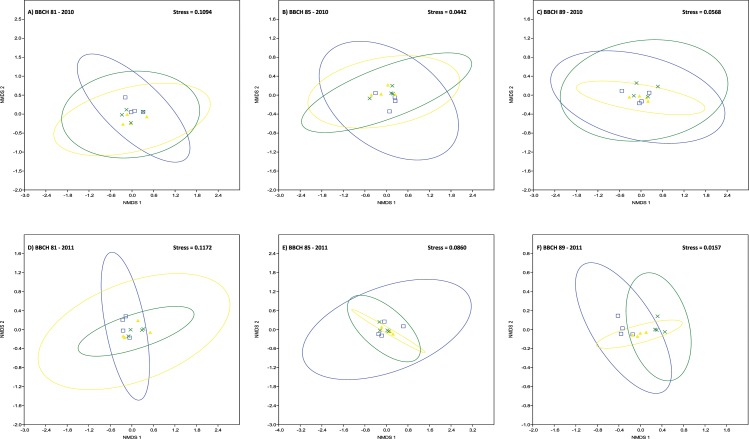
Non-metric multidimensional scaling (NMDS) plot of the fungal OTU-based clustering data. The clustering of samples in NMDS ordination indicates that fungal composition is similar between vineyard management systems (blue squares, blue ellipse = conventional plots; yellow triangles, yellow ellipse = organic plots; green crosses, green ellipse = biodynamic plots). Symbols represent sample values with 95% confidence ellipses drawn around the group centroid. Samples were collected in 2010 (A-C) and 2011 (D-F), respectively, at three different stages of grape maturation (A, D: BBCH 81; B, E: BBCH 85; C, F: BBCH 89).

Grape cluster carposphere fungal communities were not affected by management system. Graphical representations of fungal community relationships based on similarities in fungal species composition by NMDS (Fig 3) showed no distinct clustering patterns of samples according to management system. Instead, overlapping 95% confidence ellipses in NMDS ordination indicated that fungal species compositions were similar between management systems (stress values of 0.0442 to 0.1094 for 2010 fungal samples, stress values of 0.0157 to 0.1172 for 2011 samples) (Fig 3). Similar results were obtained for an analysis of similarities (ANOSIM) of fungal communities in samples obtained in 2010 from the different plots at the same maturation stage, which showed predominantly R values under 0.5 indicating that the samples are barely separable and that there is thus little or no effect of the respective management system (S4 Table).

**Fig 4:**
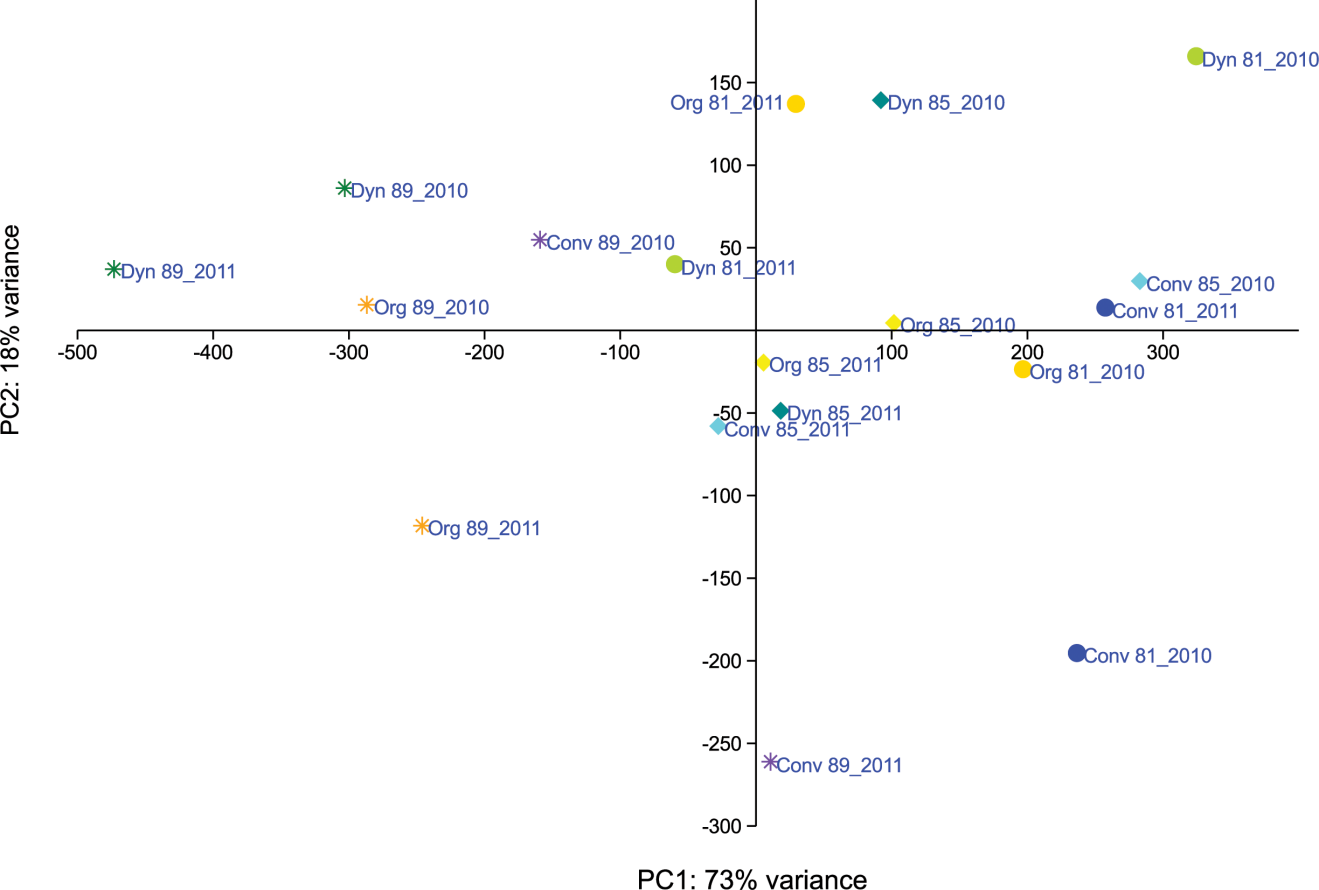
Principal component analysis (PCA) of obtained fungal OTUs present in the carposphere of grapes. Samples were collected in 2010 and 2011, respectively, at three different stages of berry maturation. The first two principal components are plotted. Colors indicate samples from different vineyard management systems (blue/purple = conventional plots; yellow/orange = organic plots; green = biodynamic plots); shapes indicate different time points (dot = BBCH 81; diamond = BBCH 85; star = BBCH 89).

With respect to shifts in fungal communities during berry ripening, in 2010, clearly separable fungal communities (R > 0.5) were evident at different maturation stages either within the same management system or on grapes from different management systems and maturation stages (S4 Table). Fungal communities on conventional grapes differed between berry maturation stages BBCH 81 and BBCH 89, just as did communities on biodynamic grapes at the same maturation stages. For 2011 grape samples, fungal communities were different (R > 0.5) or clearly separated (R > 0.75) in all grape samples by management system between maturation stages BBCH 81 and BBCH 89 as well as between BBCH 85 and BBCH 89 (printed in bold in S4 Table). Moreover, on berries ripe for harvest (BBCH 89) in 2011 fungal communities were different or clearly separated on conventional, organic and biodynamic grapes, respectively (S4 Table). The dataset obtained for fungal communities in both years were further analysed by PCA (Fig 4). The first two principal components (PCs) explained 73 and 18% of the variation, respectively. The first PC separated grape fungal communities at the stage BBCH 89 (berries ripe for harvest) from communities present at earlier stages of berry ripening (BBCH 81 and 85).

The ecological diversity of fungal communities was estimated using both the Simpson and Shannon diversity indices as well as Chao1 richness estimator. Values ranged from 0.422 to 0.7793 (2010 data) and 0.271 to 0.808 (2011 data) for the Simpson index, from 0.9373 to 1.873 (2010 data) and 0.555 to 1.982 (2011 data) for the Shannon diversity index and from 10 to 30 (2010 data) and 5 to 31 (2011 data) for Chao1 richness estimator, respectively (S5 Table). Tukey HSD test or Kruskal-Wallis test (both at α = 0.05) was used to test for significant differences between obtained diversity indices and richness estimator numbers. For fungal communities on berries ripe for harvest (BBCH 89), both the Shannon (*P* = 0.0000) and Simpson indices (*P* = 0.0000) as well as Chao1 richness estimator (*P* = 0.0004) were significantly higher in samples collected in 2010 than in samples collected in 2011, pointing to a higher diversity and richness of fungal communities on 2010 grape berry samples.

No significant differences in diversity indices or richness were detected among fungal communities from conventional, organic or biodynamic grapes in 2010 at all three maturation stages. However, at BBCH 89 in 2011 the Simpson diversity index was significantly higher for grape fungal communities in biodynamic plots compared to conventional grapes (*P* = 0.000233).

### Bacterial communities in the grape carposphere

A total of 46,099 bacterial 16S sequences (including 15,548 singletons) passed the quality control. The total number of reads obtained per year (without singletons) was 15,448 in samples obtained in 2010 and 15,103 in samples collected in 2011, respectively. In 2010, 6,531 bacterial OTUs were identified on grape samples from the biodynamic plot, while 4,744 OTUs were obtained from samples of the conventional and 4,173 OTUs from samples of the organic vineyard plot. In 2011, 7,186 OTUs were identified on biodynamic grapes, 5,646 OTUs on organic and 2,271 OTUs on conventional grapes, respectively.

Rarefaction curves at 5% dissimilarity reached an asymptote and were close to saturation, indicating that the number of individual species being sequenced was acceptable (S4 Fig). For a few samples obtained in 2011 rarefaction curves reached a plateau at a very low number of bacterial genera, which might be due to a failure of sequencing reactions for two samples obtained from two organic vineyard plots at BBCH 85.

**Fig 5:**
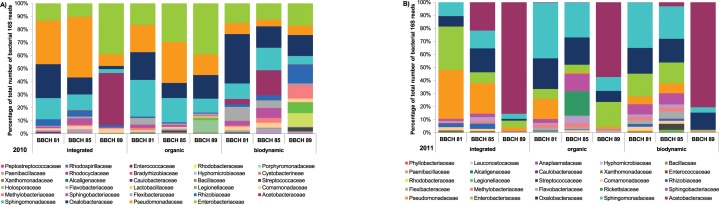
**Taxonomic distribution of bacterial communities in the carposphere of conventional, organic and biodynamic grape clusters in 2010 (A) and 2011 (B).** The distribution of the reads indicates the number of OTUs in each bacterial family based on 16S sequence data. Grapes were sampled at three stages of berry maturation (BBCH 81, BBCH 85 and BBCH 89). Only bacterial families contributing to at least 0.2% of the obtained reads are shown.

**Fig 6:**
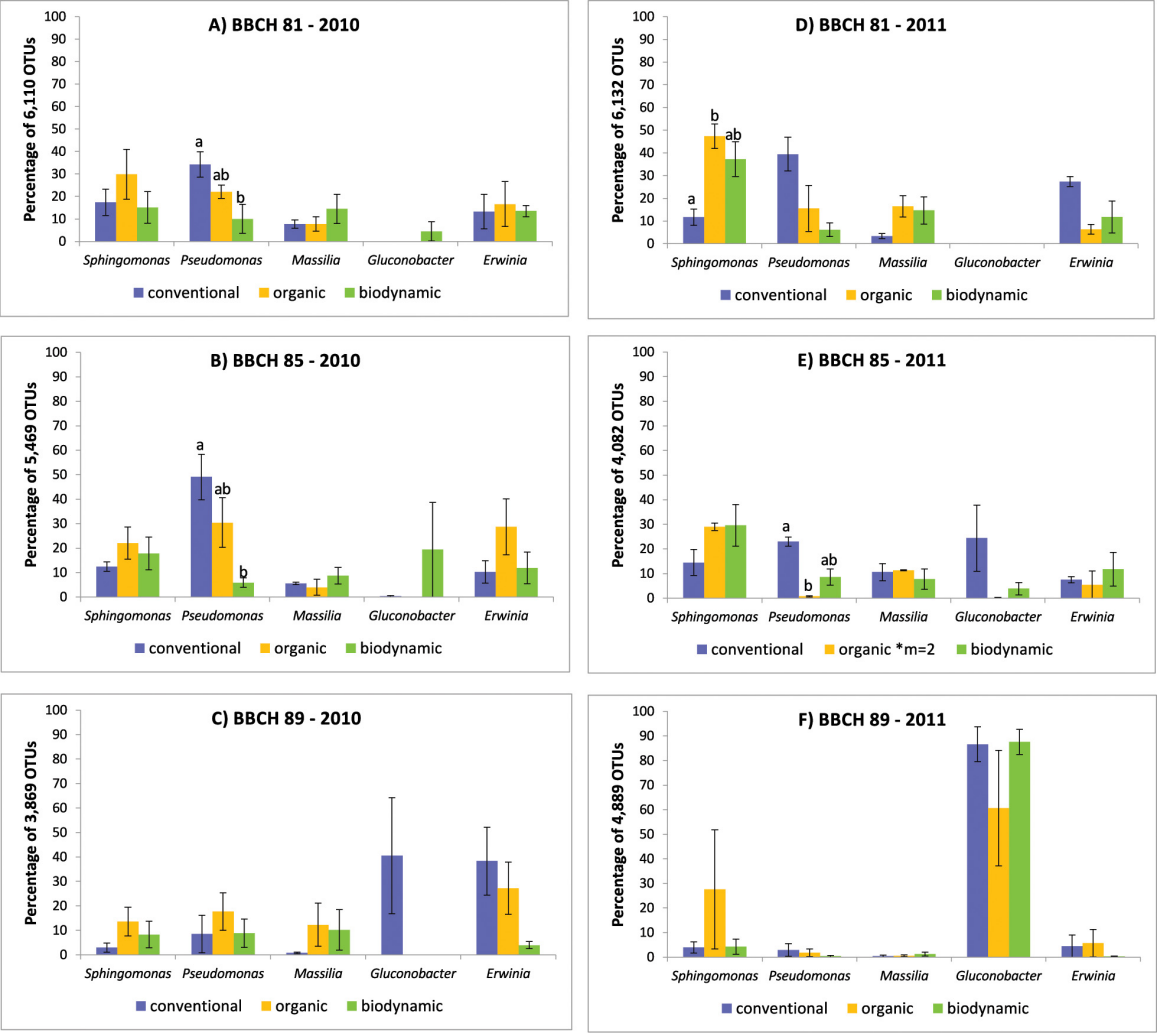
Percentage of identified OTUs belonging to the five most abundant bacterial genera. Grapes were sampled in conventional (blue), organic (yellow) and biodynamic (green) vineyard plots in 2010 (A-C) and 2011 (D-F), respectively, at three different stages of maturation (A, D: BBCH 81, beginning of ripening; B, E: BBCH 85, softening of berries; C, F: BBCH 89, berries ripe for harvest). Bars indicate standard errors of four pooled grape samples. Significant differences between abundance of individual bacterial genera and management systems within the same year and stage of berry ripening are indicated by different letters (Kruskal-Wallis test, *P* < 0.05).

To identify the most frequent bacterial taxa present in grape carposphere and rachis samples, OTUs were clustered with all obtained reads. Taxonomic distribution of bacterial 16S data based on proportions of OTUs at the family level is shown in Fig 5. The five most abundant OTUs at genus level (5% sequence dissimilarity) present in both sampling years represented 67% of the total number of reads in 2010 samples and 78% in 2011 samples, respectively (S6 and S7 Tables; Fig 6). Among them were in particular members of the genus *Sphingomonas*, followed by *Gluconobacter* (among others acetic acid bacteria), *Pseudomonas*, *Erwinia*, and *Massilia* (Fig 6).

Obtained bacterial OTUs were analyzed regarding statistical differences in the structural composition of bacterial biota present in the carposphere of grape clusters. The overall abundance of *Gluconobacter* spp. in the grape carposphere from all three management systems was significantly higher in 2011 than in 2010 (*P* = 0.0210 for BBCH 85; *P* = 0.000 for BBCH 89). The overall abundance of *Sphingomonas* spp., *Pseudomonas* spp., *Erwinia* spp. and *Massilia* spp. decreased during the ripening periods in both years (Fig 6) and was significantly higher in 2010 than in 2011 at BBCH 89 (*Pseudomonas* spp.: *P* = 0.0464; *Massilia* spp.: *P* = 0.0327; *Erwinia* spp.: *P* = 0.0061). Abundance of *Pseudomonas* spp. in the carposphere of grape clusters was significantly higher on berries obtained from conventional plots compared to berries from biodynamic plots sampled in 2010 at BBCH 81 (*P* = 0.0427) and BBCH 85 (*P* = 0.0323) (Fig 6). In addition, at BBCH 85 in 2011, significantly more *Pseudomonas* spp. were present on conventional compared to organic berries (*P* = 0.0228). In contrast, *Sphingomonas* spp. were significantly less abundant on conventional compared to organic berries at BBCH 81 in 2011 (*P* = 0.0427).

**Fig 7:**
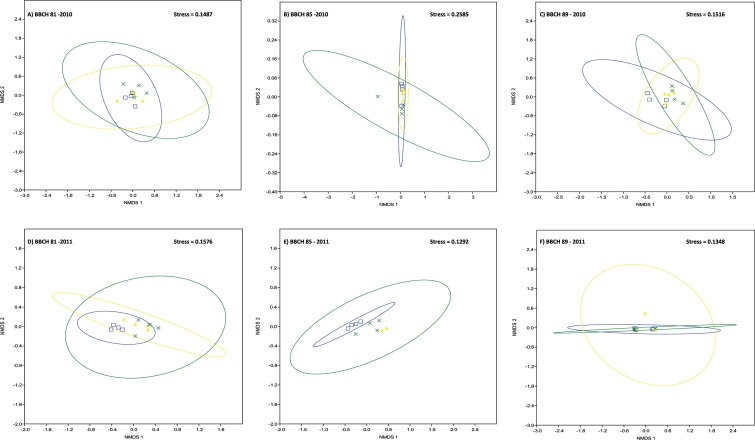
Non-metric multidimensional scaling (NMDS) plot of the bacterial OTU-based clustering data. The clustering of samples in NMDS ordination indicates that bacterial composition is similar between vineyard management systems (blue squares, blue ellipse = conventional plots; yellow triangles, yellow ellipse = organic plots; green crosses, green ellipse = biodynamic plots). Symbols represent sample values with 95% confidence ellipses drawn around the group centroid. Samples were collected in 2010 (A-C) and 2011 (D-F), respectively, at three different stages of grape maturation (A, D: BBCH 81; B, E: BBCH 85; C, F: BBCH 89).

Bacterial communities in the grape cluster carposphere were not affected by management system. Graphical representations of bacterial community relationships based on similarities in bacterial species composition by NMDS (Fig 7) showed no distinct clustering patterns of samples according to management system (stress values of 0.1487 to 0.2585 for 2010 samples; stress values of 0.1292 to 0.1576 for samples collected in 2011) (Fig 7). Likewise, analysis of similarities (ANOSIM) of bacterial samples obtained from the carposphere of grape clusters collected in 2010 showed predominantly R values under 0.5 indicating that the samples are barely separable and that there is thus little or no effect of the applied management system (S8 Table). With respect to shifts in bacterial communities during berry ripening, in 2010, clearly separable bacterial communities (R > 0.5) were evident at different maturation stages either within the same management system (grapes from conventional and biodynamic plots, respectively, sampled at BBCH 81 and BBCH 89) or on grapes from different management systems and maturation stages (S8 Table). Bacterial communities on conventional grapes differed between berry maturation stages BBCH 81 and BBCH 89, as did communities on biodynamic grapes at the same maturation stages. In 2011, bacterial communities were different (R > 0.5) or clearly separated (R > 0.75) in all grape samples from the conventional vineyard plots between maturation stages BBCH 81 and BBCH 89 as well as between BBCH 85 and BBCH 89 (typed in bold in S8 Table). Moreover, in 2011 bacterial communities present on grapes at the beginning of ripening (maturation stage BBCH 81) were different or clearly separated between conventional and organic as well as between conventional and biodynamic grapes, respectively (S8 Table). An absolute separation (R = 1) was observed between grape samples collected from the conventional and organic plots at BBCH 85. However, this separation could be caused by failure of the sequencing reaction for two samples obtained from the organic plot at BBCH 85.

**Fig 8:**
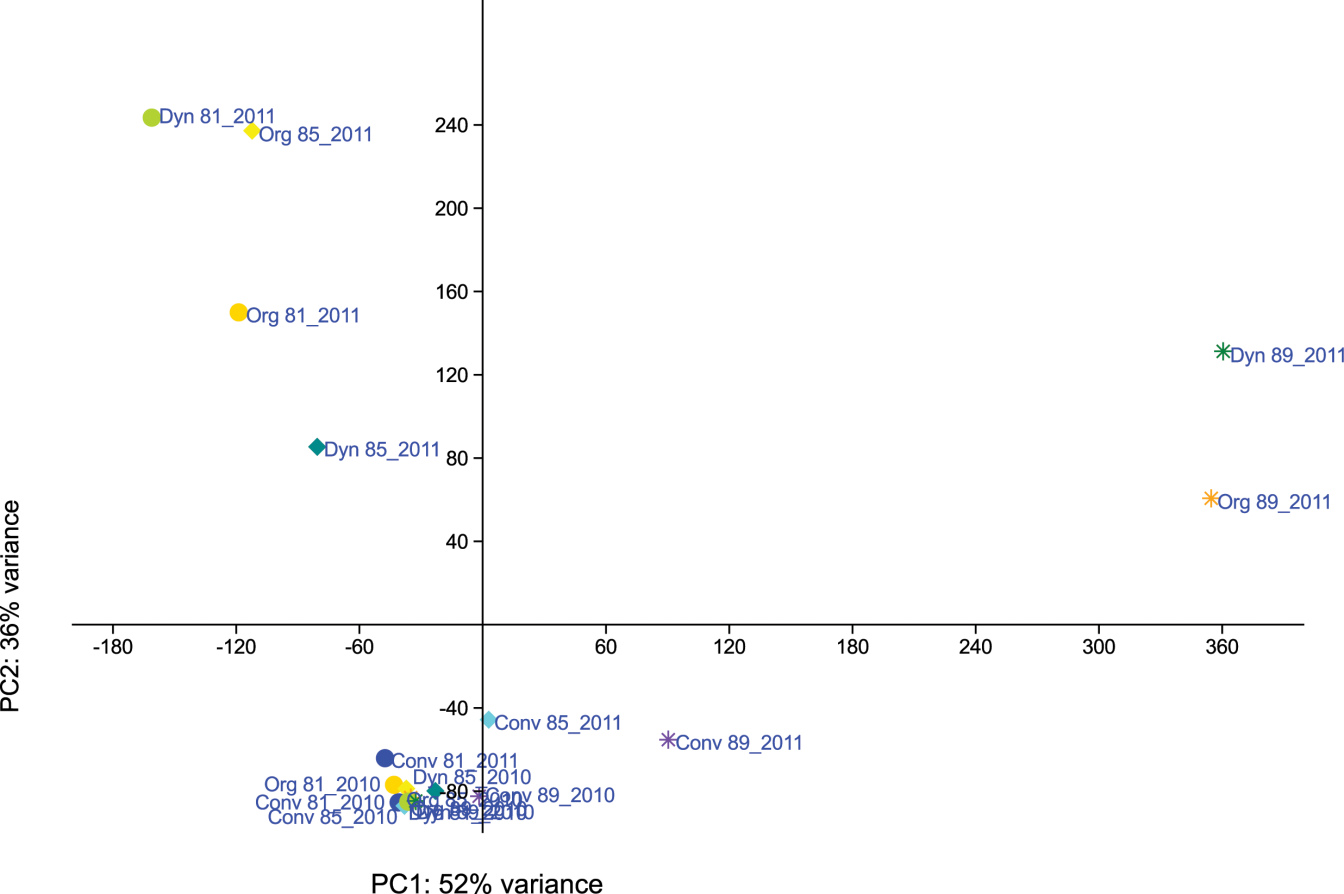
Principal component analysis (PCA) of obtained bacterial OTUs present on the carposphere of grapes. Samples were collected in 2010 and 2011, respectively, at three different stages of berry maturation. The first two principal components are plotted. Colors indicate samples from different vineyard management systems (blue/purple = conventional plots; yellow/orange = organic plots; green = biodynamic plots); shapes indicate different time points (dot = BBCH 81; diamond = BBCH 85; star = BBCH 89).

The dataset obtained for bacterial communities in both years were further analysed by PCA (Fig 8). The first two principal components explained 52 and 36% of the variation, respectively. The second PC separated grape bacterial communities at the stage BBCH 89 (berries ripe for harvest) from communities present at earlier stages of berry ripening (BBCH 81 and 85).

The ecological diversity of bacterial communities was estimated using the Simpson and Shannon diversity indices as well as in Chao1 richness estimator. Values ranged from 0.3402 to 0.92983 (2010 data) and 0.000 to 0.9143 (2011 data) for the Simpson index, from 0.8540 to 3.0170 (2010 data) and 0.000 to 2.777 (2011 data) for the Shannon diversity index and from 10 to 50.5 (2010 data) and 1 to 43 (2011 data) for Chao1 richness estimator, respectively (S9 Table). Similar to results obtained for fungal communities on berries ripe for harvest (BBCH 89), both the Shannon (*P* = 0.0013) and Simpson indices (*P* = 0.0027) as well as Chao1 richness estimator (*P* = 0.0261) were significantly higher for bacterial communities on samples collected in 2010 than on samples collected in 2011, pointing to a higher diversity and richness of bacterial communities on 2010 grape berry samples. No significant differences in diversity indices or richness were detected among bacterial communities from conventional, organic or biodynamic grapes sampled in 2010 as well as in 2011 at all three maturation stages.

### Relative quantification of potential fungal antagonists

**Fig 9:**
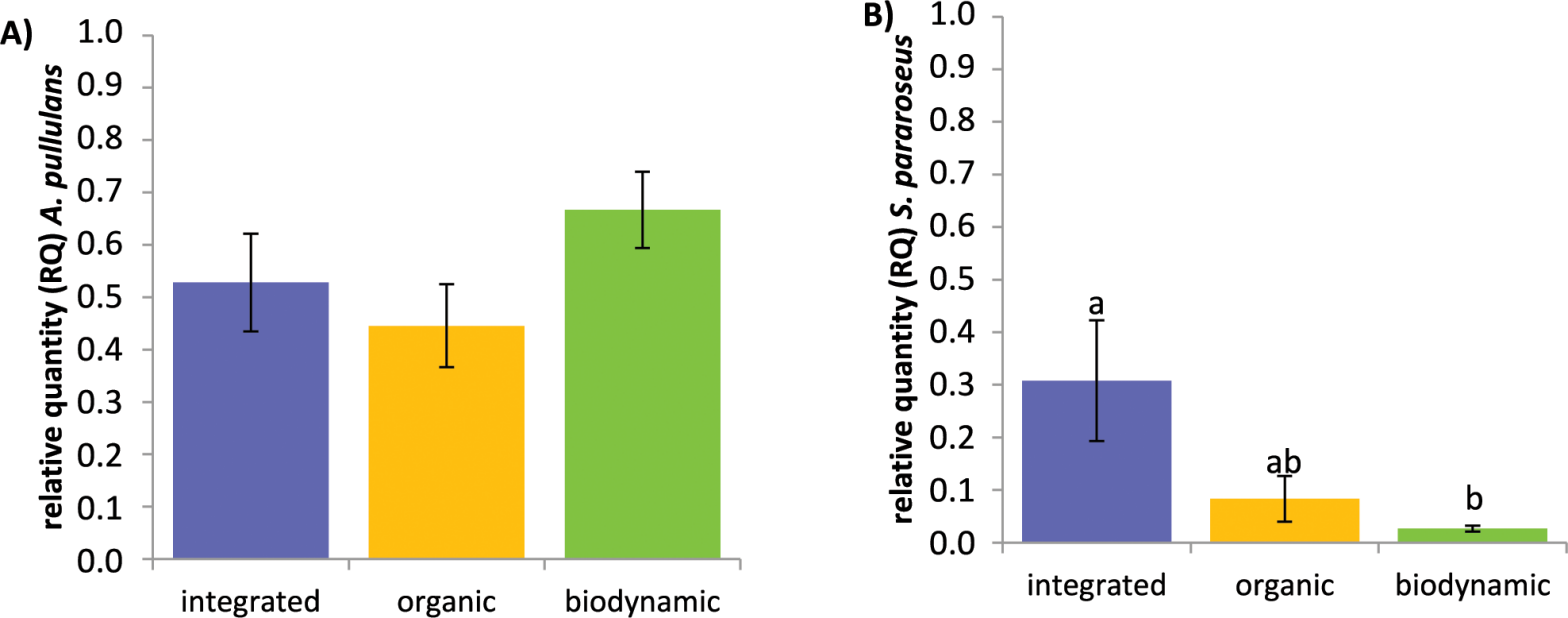
**Relative DNA amounts of *A*. *pullulans* (A) and *S*. *pararoseus* (B) DNAs on grape berry samples.** Grapes were obtained from conventional (blue), organic (yellow) and biodynamic (green) vineyard plots at the beginning of berry ripening (BBCH stage 81) in 2010. Significant differences were found regarding the relative amount of *S*. *pararoseus* DNA on conventional and biodynamic grape berries (Kruskal-Wallis test; *P* < 0.05) and are indicated by different letters.

A relative quantification of *S*. *pararoseus* and *A*. *pullulans* ITS copy numbers at the beginning of berry ripening in samples obtained in 2010 was assessed via qPCR and was normalized to the relative quantity of the geometric mean of the relative quantities of two fungal reference genes (actin and ITS). For *S*. *pararoseus*, a significantly higher amount (*P* = 0.0027) was found in berry samples obtained from the conventional vineyard plots compared to those from the biodynamic ones (Fig 9). *A*. *pullulans* tended to be present in slightly higher yet non-significant relative amounts (*P* = 0.1705) in the carposphere of grapes cultivated in biodynamic vineyard plots, than in the conventionally or organically managed plots.

## Discussion

In our study on the composition of microbial communities on grape berries during the period of berry ripening, viticultural management system (conventional, organic or biodynamic) had no significant effects on the abundance, diversity and richness of fungi or bacteria present in the grape carposphere. Sampling of berries was performed in two subsequent years and at three stages during berry maturation in a vineyard containing plots with all three management systems cultivated side by side for several years before initiation of the study. Thus, our study design does exclude any influences on composition of microbial communities e.g. due to grapevine age, rootstock, different soil attributes, or other abiotic factors. Only a few studies have been conducted so far on the effect of viticultural management systems on microbial communities present on grape berries [24, 42, 43], albeit such data are of high relevance in order to harvest and process high quality grapes.

### Shift in abundance of microorganisms during berry ripening

Fungal communities found in the carposphere of cv. Riesling grape berries predominately consisted of members of the families *Sclerotiniaceae* (in particular *B*. *cinerea*), *Davidiellaceae* (e.g. *Cladosporium* spp., *Davidiella tassiana*), *Dothioraceae* (e.g. *A*. *pullulans*), *and Pleosporaceae* (e.g. *A*. *alternata*). Members of these fungal families also dominated the fungal community on cv. Chardonnay and cv. Cabernet Sauvignon grape berries [44]. Most common bacterial families present in the carposphere of grapes in our study were *Acetobacteraceae*, *Enterobacteraceae*, *Sphingomonadaceae*, *Oxalobacteraceae*, and *Pseudomonadaceae* which is in accordance with the results obtained by Bokulich et al. [44] regarding composition of bacterial communities on grape berries.

Abundance of members of *Sclerotiniaceae* (mainly *B*. *cinerea*), the most common fungal family found in this study, increased in both years during the ripening period independent of the management system. In contrast, the abundance of all other fungi e.g. *Cladosporium* spp., *A*. *pullulans* and *A*. *alternata* decreased. *B*. *cinerea*, *Cladosporium* spp., and *A*. *alternata* are well known as the most common fungal mould organisms isolated from grapes [45]. The same was observed regarding the development of bacterial communities during berry ripening. The abundance of members of the *Acetobacteraceae* (e.g. *Gluconobacter*–acetic acid bacteria) increased in both years during the ripening period independent of the management system, while the abundance of all other bacteria e.g. *Massilia* spp., *Erwinia* spp., *Pseudomonas* spp. and *Sphingomonas* spp. decreased. Fungi and bacteria cohabitate the surface of grape berries, thus they compete for example for the acquisition of nutrients like sugar. Leaks in the grape berry skin during ripening increase the accessibility to sugar allowing some microorganisms to establish and multiply more efficiently than others thereby altering the composition of berry microbiota [3]. Apparently, such a selective advantage allowed *B*. *cinerea* and acetic acid bacteria to grow faster during the ripening period of grape berries, and suppressing but not completely eliminating the growth and abundance of other microorganisms.

Overall, our observed shifts in abundance of certain microorganism during the berry ripening process are in accordance with results from other studies on the grapevine microbiome. On grapevine leaves, the major abundant microorganisms during the vegetative cycle were the yeast-like fungus *Aureobasidium* spp. and members of the prokaryotic Enterobacteriaceae [46]. In the same study, microbial communities on grapevine leaves were shown to be highly structured and dynamically changed along the vegetative cycle [46]. Moreover, an increase in the yeast and yeast-like populations throughout the berry ripening process have been recently documented by culture independent methods [42].

### Vintage effect

As we performed our study in the same vineyard in two subsequent years, an effect of vintage on composition of microbial communities was detectable. Both, fungal and bacterial diversity were significantly higher in 2010 than in 2011 at the last stage of berry ripening. Accordingly, the four most abundant fungal species as well as four of the five most abundant bacterial species were significantly more prevalent on ripe berries sampled in 2010 than in 2011. In a study of the microbiota of the grapevine phyllosphere Perazzolli et al. [43] also observed a clear influence of the field site on abundance of certain groups of bacteria and fungi, and were also able to prove a significant correlation to the prevailing environmental parameters of the respective location such as temperature and rainfall. In line with that, a significant impact of vintage and thus specific climatic features on the composition of grape must microbiota was detected in a recent study performed by Bokulich et al. [44].

**Fig 10:**
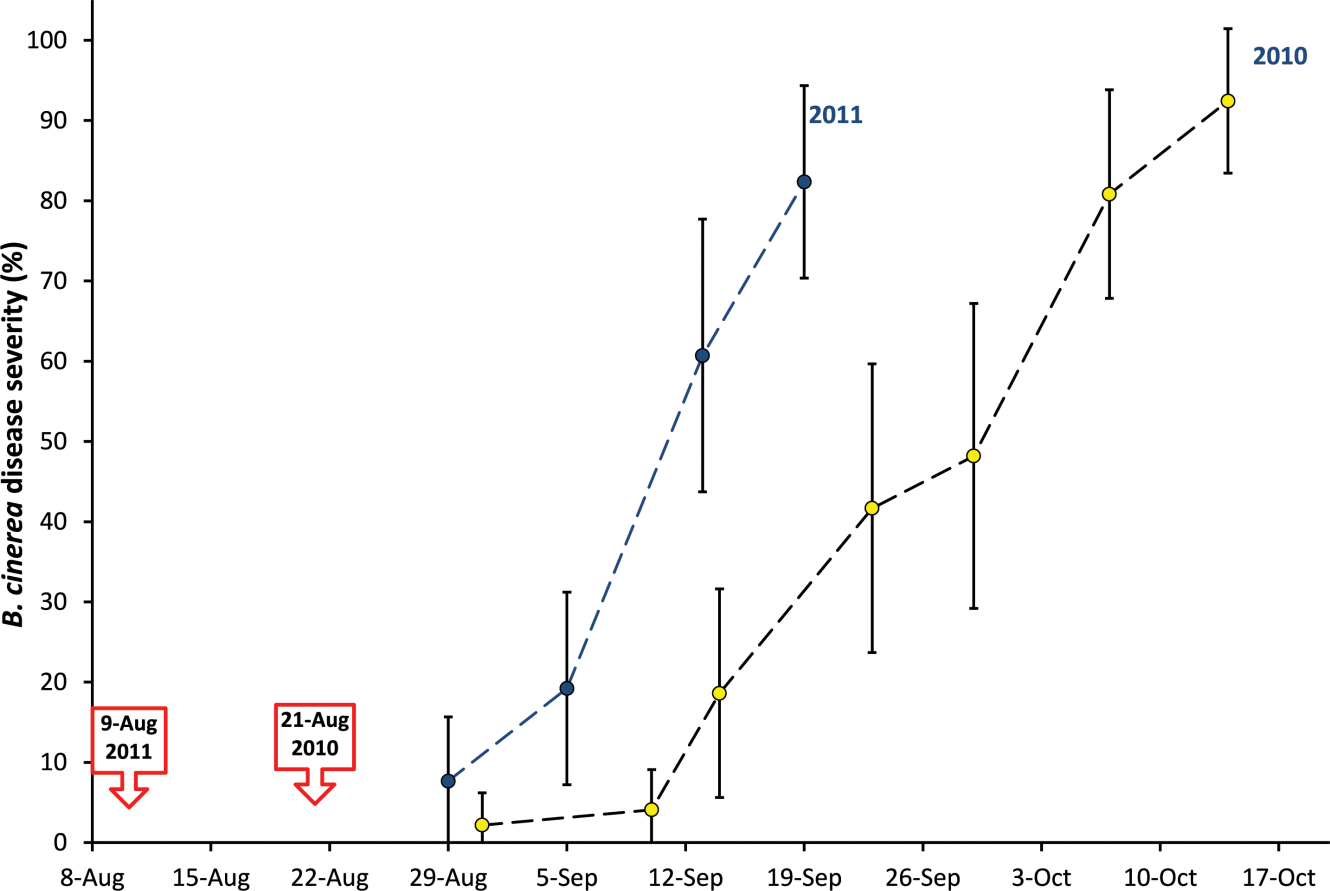
*Botrytis cinerea* disease severity (%) in an untreated vineyard plot in 2010 and 2011 in Geisenheim, Germany. 100 bunches of cv. Riesling grapes were assessed weekly for *B*. *cinerea* infections. Lines indicate standard deviations. Beginning of grape ripening in 2010 and 2011 is marked with a red arrow.

Climatic conditions prevailing during the berry ripening and sampling period in late summer and autumn 2010 and 2011, respectively, were not considerably different. With average monthly temperatures of 18.0°C and 13.7°C, respectively, August and September 2010 were only slightly cooler than the same months in 2011 (19°C for August and 16.6°C for September 2011, respectively). Moreover, August and September 2011 had less precipitation than 2010 (sum of 177 mm in August and September 2010 and 144 mm in August and September 2011, respectively). However, 2011 was a year with an early start of grape ripening (9^th^ of August 2011 compared to 21^st^ of August in 2010) and an accordingly earlier onset and a more rapid progression of *Botrytis* infections as can be seen in a vineyard not treated with fungicides against *B*. *cinerea* and neighboring to the experimental vineyard used for sampling grapes in the present study (Fig 10).

### Management effect

Overall, there was no distinct fungal or bacterial community associated with berries sampled from conventional, organic or biodynamic vineyard plots in our study. Exceptions were fungal communities present on berries ripe for harvest (last stage of berry ripening) in 2011, which were different or clearly separated between grapes from the three management systems. In addition, no significant differences in diversity or richness of fungal or bacterial communities were detected on grapes from the three different management systems in both subsequent years of sampling. This finding is in agreement with a recent study by Perazolli et al. [43], where bacterial and fungal communities present in the phyllosphere of untreated grapevine leaves or on leaves treated either with a biocontrol agent or a synthetic fungicide did not significantly differ from each other. However, both fungal as well as bacterial endophytic communities were different in grapevines from organic compared to conventional vineyards [38, 47]. Moreover, using single-strand conformation polymorphism analysis (SSCP) Schmid et al. [24] and Grube et al. [48] have shown that plant protection strategies applied in conventional and organic vineyards influenced structure and function of grape-associated microorganisms in samples of leaves, shoots and grapes. In these studies, this difference was due in particular to the dominance of *A*. *pullulans* which was strongly enriched in communities of organically managed grapevines. *Aureobasidium* spp. was also more frequently associated with organic than with conventional grapes in a study performed by Martins et al. [42]. This yeast-like (black) fungus is a well-known potential antagonist of *B*. *cinerea* or *Aspergillus carbonarius*, one of the causal agents of sour rot of grape berries. *Aureobasidium* spp. also is known to tolerate copper and sulphur applications and is well adapted to the phyllosphere and carposphere of various plants [3, 49–52]. Yet, in our study, there was no significant difference in the abundance of *A*. *pullulans* on conventional, organic or biodynamic grapes (as revealed by both pyrosequencing and qPCR data). However, on ripe berries *A*. *pullulans* tended to be present in a slightly higher amount in the carposphere of conventional grapes compared to organic or biodynamic grapes (pyrosequencing data). Accordingly, vineyard specific characteristics, grape variety, prevailing abiotic conditions or biotic interactions on the grape berry might have a stronger effect on abundance of *A*. *pullulans* on grapes than the vineyard management system (conventional, bioorganic or biodynamic viticulture) itself. Yet, this assertion requires further examinations in various climates and grapevine growing regions.

A preliminary study albeit based on a small sample size by Leveau and Tech [53] has indicated that composition of bacterial communities on grape berries are different from those found on grape leaves. Thus, in the context of understanding microbial disease progression and ecology in vineyards under different management systems, a challenge for future studies would be a simultaneous analysis of microbial communities present on both leaves and berries and their temporal and spatial shifts in species composition.

A second potential antagonist of *B*. *cinerea*, *Sporidiobolus pararoseus*, a red pigmented yeast associated with grapes and known to produce toxins [22, 54], was found in a significantly higher abundance in the carposphere of grapes cultivated in conventional compared to biodynamic vineyards (qPCR data). This is in accordance with results reported by both Schmid et al. [24] and Martins et al. [42], where significantly larger amounts of *S*. *pararoseus* were detected on berries and in endosphere samples of conventional grapes compared to organic ones. In the vineyard plots used for our study as well as in the studies by Schmid et al. [24] and Martins et al. [42], copper was applied in the organic and biodynamic vineyard plots but not in the conventional plots. Copper is frequently applied in organic vineyards to control grapevine diseases [55], has accumulated in vineyard soils worldwide and is known to be toxic for various microorganisms [56]. Accordingly, it could be postulated that the lower abundance of *S*. *pararoseus* in organic compared to conventional vineyards is due to a negative effect of copper. Synthetic fungicides applied in conventional viticulture can also select for specific microorganisms. However, these postulations require additional laboratory studies.

In 2010 and 2011, disease severity of *Botrytis* bunch rot was assessed in the same vineyard plots used for the present study (for data see [31]) and was shown to be significantly increased in the biodynamic compared to the conventional vineyard plots. However, there were no significant differences in disease severity of *B*. *cinerea* between the conventional and organic plots, as well as between organic and biodynamic plots, respectively [31]. Accordingly, neither the application of synthetic botryticides nor the application of biodynamic preparations can solely account for these observations. The study of Döring et al. [31] in the same vineyard as the one assessed here also revealed higher nitrogen levels both in the soil and in leaf tissues sampled in organic and biodynamic vineyard plots compared to those obtained from conventional plots. Higher nitrogen levels are known to increase vine vigour and canopy density, thereby increasing the infection risk by *B*. *cinerea* [57]. Moreover, nitrogen levels in the soil might as well influence belowground microbial communities. In this regard, Zarraonaindia et al. [58] have shown that soil serves as a key source of grapevine-associated bacteria, thus any differences in soil management and soil characteristics as expected under different management systems might as well influence microbiota present in the grape carposphere.

## Conclusions

Generally, composition of microbial communities present in the carposphere of ripening grape clusters did not differ between grapes produced in conventional, organic, and biodynamic plots. Exceptions were composition of fungal communities present on ripe berries in 2011 and abundance of particular species like *A*. *pullulans* or *A*. *alternata*, where significant differences were evident at certain stages during berry ripening and only in one of the two years of sample analysis. Shifts in composition of microbial communities on the surface of the grape berry skin were attributed to progression of berry ripening or to a vintage effect in the two respective years of this study. Thus our study has shown that management systems like conventional or organic viticulture tend to influence the abundance of certain microorganisms rather than causing shifts in complete microbial communities. Microorganisms can contribute both to grape health in the vineyard as well as to many important processes during wine making like malolactic fermentation or wine spoilage. Moreover, species like *A*. *pullulans* or *S*. *pararoseus* present on grape berries can serve as effective indigenous antagonists against microorganisms known to have negative effects on wine quality such as *B*. *cinerea*. Respective vineyard management system or certain viticultural practices manipulating the presence or absence of these organisms thus represent important strategies for a sustainable grape production in the field.

## Supporting Information

S1 Fig Map of the vineyard used for sampling of grapes in the present study in 2010 and 2011. (DOCX)

S2 Fig Climatic conditions 2010, obtained from a weather station of the German meteorological service DWD (Deutscher Wetterdienst) located next to the experimental vineyard in Geisenheim. (PDF)

S3 Fig Rarefaction curves illustrating the observed number of fungal species in grape samples obtained from conventional, organic and biodynamic vineyard plots in 2010 and 2011. (DOCX)

S4 Fig Rarefaction curves illustrating the observed number of bacterial genera in grape samples obtained from conventional, organic and biodynamic vineyard plots in 2010 and 2011. (DOCX)

S1 Table Plant protection agents applied to the conventional, organic and biodynamic vineyard plots during the years 2006–2011. (DOCX)

S2 Table Number of obtained fungal reads (OTU at species level without singletons) obtained from conventional, organic and biodynamic grapes sampled at three maturation stages in 2010. (XLSX)

S3 Table Number of obtained fungal reads (OTU at species level without singletons) obtained from conventional, organic and biodynamic grapes sampled at three maturation stages in 2011. (XLSX)

S4 Table Computation of R values after analysis of similarities between fungal samples obtained from conventional, organic and biodynamic grapes sampled at three different stages of berry maturation. R values obtained for 2010 samples are shown below the diagonal, R values for 2011 samples are printed in italics above the diagonal.
(DOCX)

S5 Table Simpson and Shannon diversity indices and Chao1 richness estimator for fungal communities present on conventional, organic and biodynamic grape berries. (XLSX)

S6 Table Number of obtained bacterial reads (OTUs at genus level without singletons) obtained from conventional, organic and biodynamic grapes sampled at three maturation stages in 2010. (XLSX)

S7 Table Number of obtained bacterial reads (OTUs at genus level without singletons) obtained from conventional, organic and biodynamic grapes sampled at three maturation stages in 2011. (XLSX)

S8 Table Computation of R values after analysis of similarities between bacterial samples obtained from conventional, organic and biodynamic grapes sampled at three different stages of berry maturation. R values obtained for 2010 samples are shown below the diagonal, R values for 2011 samples are printed in italics above the diagonal.
(DOCX)

S9 Table Simpson and Shannon diversity indices and Chao1 richness estimator for bacterial communities present on conventional, organic and biodynamic grape berries. (XLSX)
