# Supplementary material for: Are Epiphytic Microbial Communities in the Carposphere of Ripening Grape Clusters (Vitis vinifera L.) Different between Conventional, Organic, and Biodynamic Grapes?
Source: PLoS One. 2016 Aug 8;11(8):e0160852. doi: 10.1371/journal.pone.0160852 (PMC4976965; doi:10.1371/journal.pone.0160852)
Supplement: S1 Fig — (DOCX) [file pone.0160852.s001.docx]

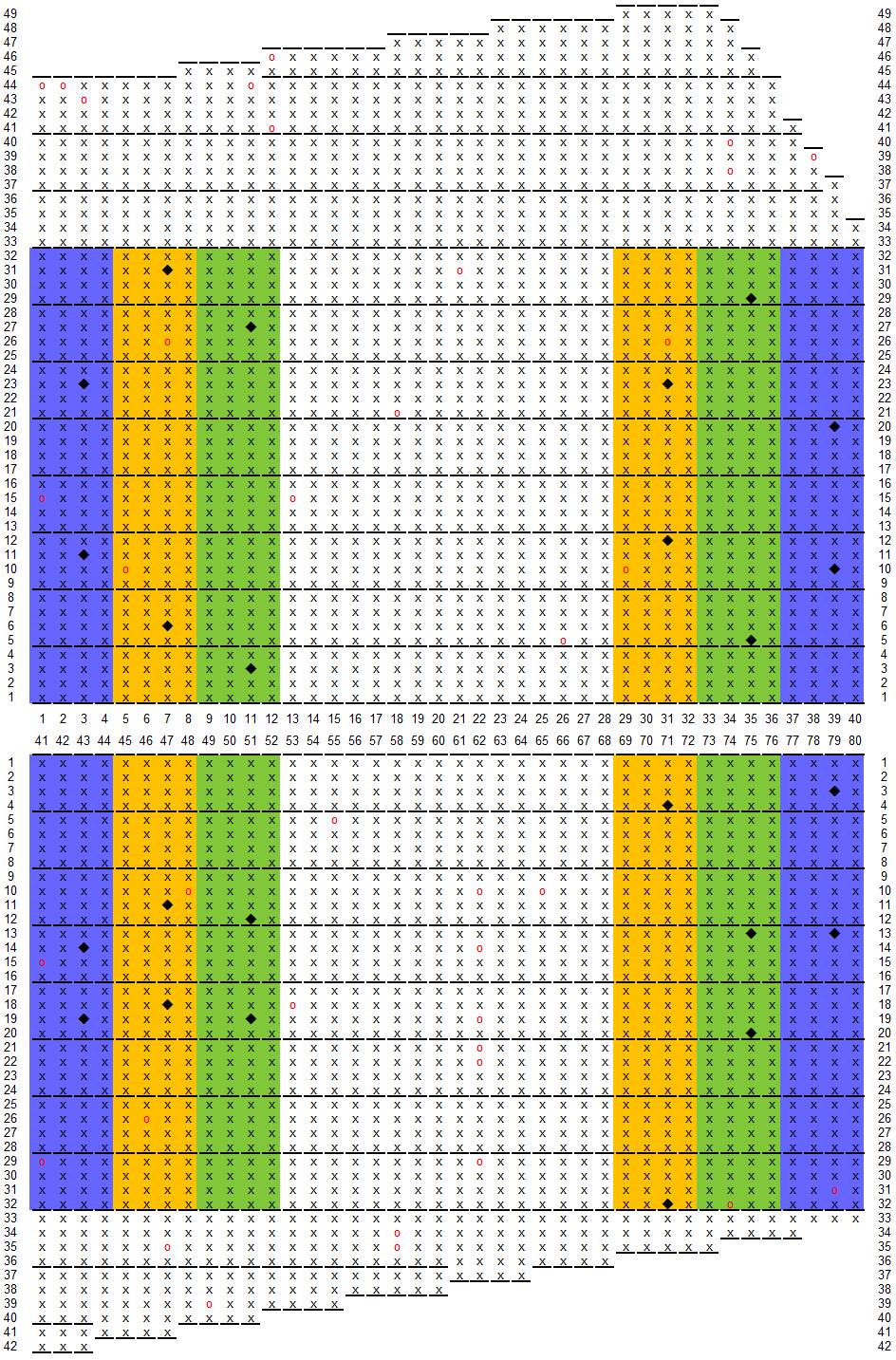


**S1 Fig.** **Map of the vineyard used for sampling of grapes in the present study in 2010 and 2011.** Plots marked in different colors indicate the applied management system practiced since 2006 (conventional in blue, organic in yellow and biodynamic viticulture in green). Crosses indicate single grapevine plants, black diamonds indicate the location of grapevine plants used for sampling.
