# Supplementary material for: Are Epiphytic Microbial Communities in the Carposphere of Ripening Grape Clusters (Vitis vinifera L.) Different between Conventional, Organic, and Biodynamic Grapes?
Source: PLoS One. 2016 Aug 8;11(8):e0160852. doi: 10.1371/journal.pone.0160852 (PMC4976965; doi:10.1371/journal.pone.0160852)
Supplement: S2 Fig — (PDF) [file pone.0160852.s002.pdf]

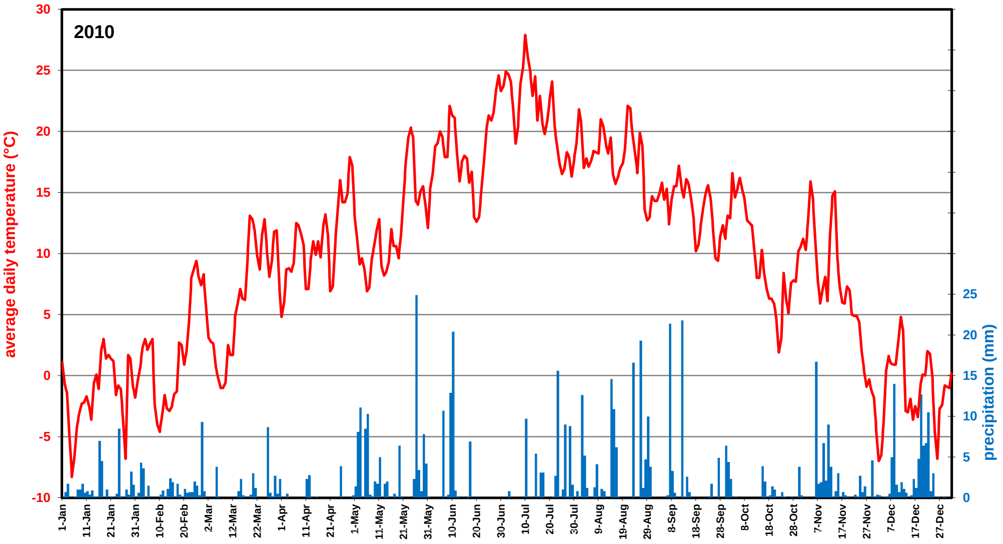

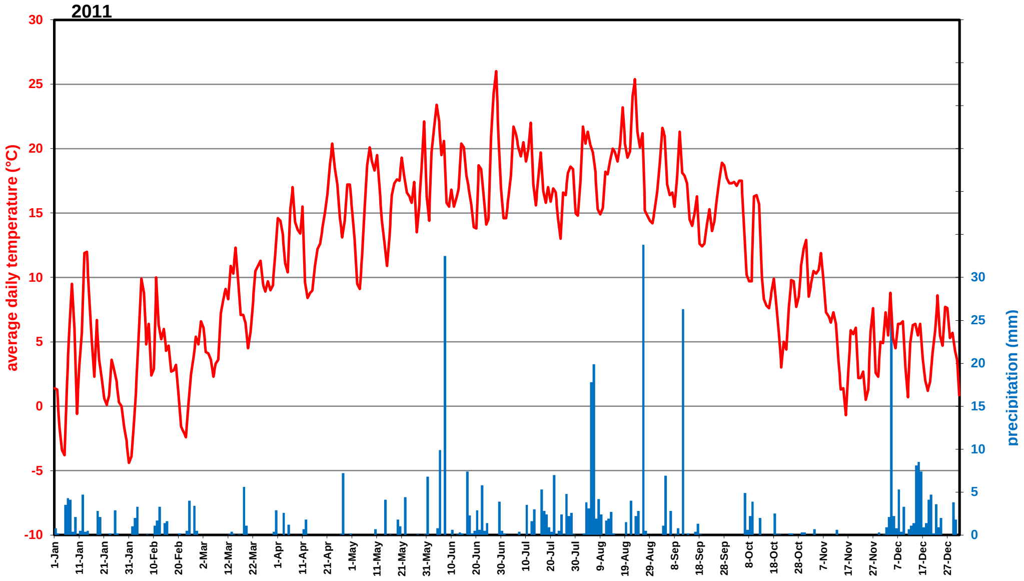

S2 Fig. Climatic conditions 2010 and 2011, obtained from a weather station of the German meteorological service DWD (Deutscher Wetterdienst) located next to the experimental vineyard in Geisenheim. The three sampling dates are marked with an arrow.
