# Supplementary material for: Are Epiphytic Microbial Communities in the Carposphere of Ripening Grape Clusters (Vitis vinifera L.) Different between Conventional, Organic, and Biodynamic Grapes?
Source: PLoS One. 2016 Aug 8;11(8):e0160852. doi: 10.1371/journal.pone.0160852 (PMC4976965; doi:10.1371/journal.pone.0160852)
Supplement: S3 Fig — (DOCX) [file pone.0160852.s003.docx]

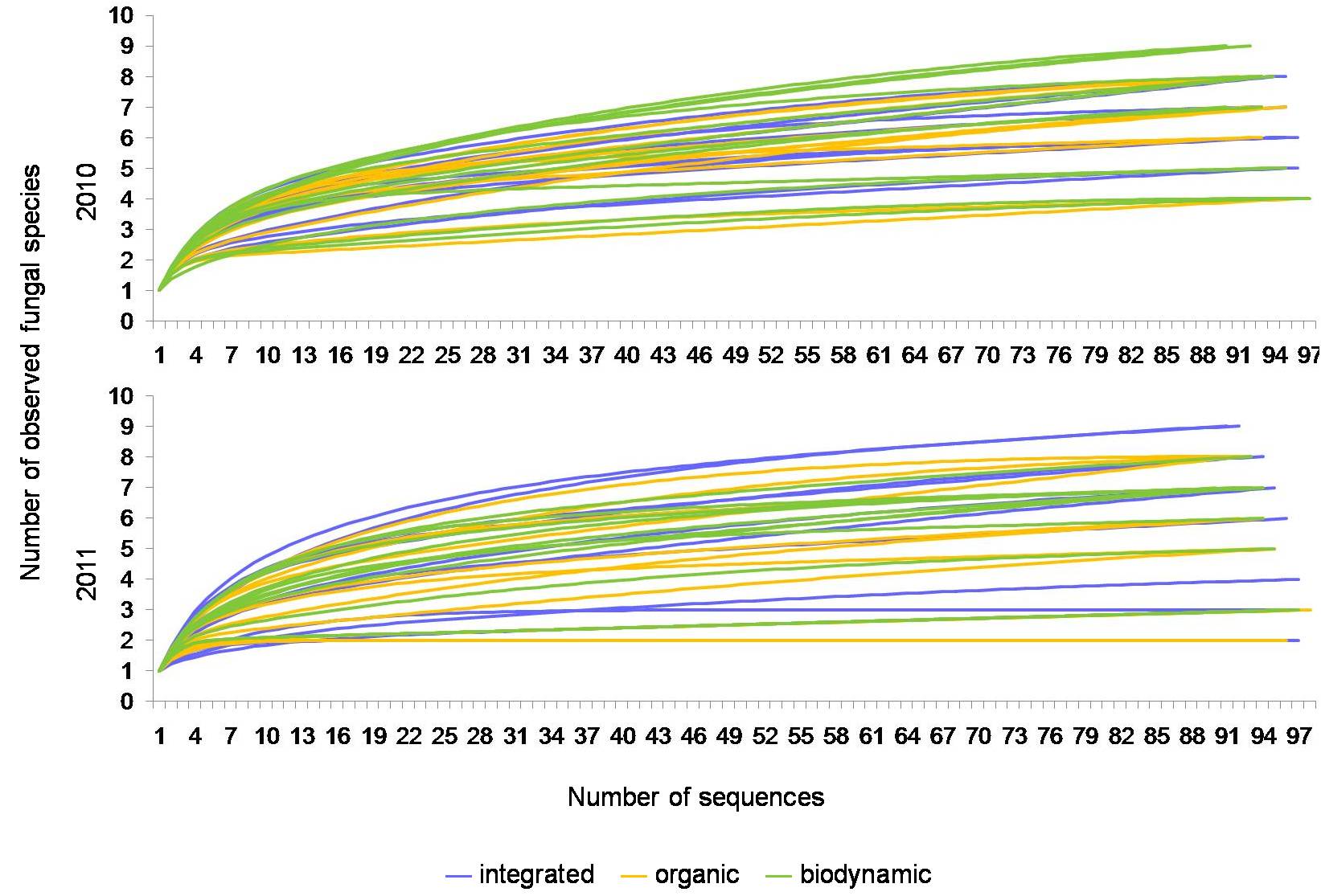


**S3 Fig. Rarefaction curves illustrating the observed number of fungal species in grape samples obtained from conventional (integrated) (blue), organic (yellow) and biodynamic (green) vineyard plots in 2010 and 2011.**
