# Supplementary material for: Are Epiphytic Microbial Communities in the Carposphere of Ripening Grape Clusters (Vitis vinifera L.) Different between Conventional, Organic, and Biodynamic Grapes?
Source: PLoS One. 2016 Aug 8;11(8):e0160852. doi: 10.1371/journal.pone.0160852 (PMC4976965; doi:10.1371/journal.pone.0160852)
Supplement: S1 Table — (DOCX) [file pone.0160852.s005.docx]

**S1 Table. Plant protection agents applied to the conventional, organic and biodynamic vineyard plots during the years 2006-2011.**

| **Conventional Vineyard Plots** | | | |
| --- | --- | --- | --- |
| **Product Name** | **Active Ingredient** | **Category** | |
| Durano | Glyphosat | herbicide | |
| Glyfos | Glyphosat |  |  |
| Netzschwefel | Sulfur | fungicide | |
| Polyram WG | Metiram |  |  |
| Forum Star | Dimetomorph + Folpet |  |  |
| Vento Power | Quinoxyfen + Myclobutanil |  |  |
| Cabrio Top | Pyraclostrobin + Metiram |  |  |
| Equation Pro | Cymoxanil + Famoxadon |  |  |
| Scala | Pyrimethanil |  |  |
| Mildicut | Cyazofamid |  |  |
| Vivando | Metrafenone |  |  |
| Folpan 80 WDG | Folpet |  |  |
| Prosper | Spiroxamine |  |  |
| Systhane 20 EW | Myclobutanil |  |  |
| Switch | Fludioxonil + Cyprodinil |  |  |
| **Organic and Biodynamic Vineyard plots** | | |  |
| **Product Name** | **Active Ingredient** | **Category** | |
| Netzschwefel | Sulfur | fungicide |  |
| Cuprozin flüssig | Copper |  |  |
| MycoSin Vin | Plant extracts, clay, yeast extracts | plant resistance inducer |  |
| Frutogard | Algae extracts, K-phosphonate |  |  |
| SaluKarb | Potassium hydrogen carbonate |  |  |
