# Supplementary material for: Are Epiphytic Microbial Communities in the Carposphere of Ripening Grape Clusters (Vitis vinifera L.) Different between Conventional, Organic, and Biodynamic Grapes?
Source: PLoS One. 2016 Aug 8;11(8):e0160852. doi: 10.1371/journal.pone.0160852 (PMC4976965; doi:10.1371/journal.pone.0160852)
Supplement: S4 Table — R values obtained for 2010 samples are shown below the diagonal, R values for 2011 samples are printed in italics above the diagonal. (DOCX) [file pone.0160852.s008.docx]

**S4 Table. Computation of R values after analysis of similarities between fungal samples obtained from conventional, organic and biodynamic grapes sampled at three different stages of berry maturation.** R values obtained for 2010 samples are shown below the diagonal, R values for 2011 samples are printed in italics above the diagonal.^a^

|  | **Conven. BBCH 81** | **Conven. BBCH 85** | **Conven. BBCH 89** | **Org. BBCH 81** | **Org. BBCH 85** | **Org. BBCH 89** | **Biodyn. BBCH 81** | **Biodyn. BBCH 85** | **Biodyn. BBCH 89** |
| --- | --- | --- | --- | --- | --- | --- | --- | --- | --- |
| **Conven. BBCH 81** |  | *0.4063* | ***1.0000*** | *0.0833* | ***0.7292*** | ***1.0000*** | *0.3229* | ***0.5208*** | ***1.0000*** |
| **Conven. BBCH 85** | -0.0833 |  | ***0.6458*** | *-0.0938* | *0.1354* | *0.4792* | *0.1146* | *-0.0729* | ***0.5000*** |
| **Conven. BBCH 89** | **0.6354** | 0.3021 |  | ***0.7708*** | ***1.0000*** | ***0.5625*** | ***1.0000*** | ***1.0000*** | ***1.0000*** |
| **Org. BBCH 81** | 0.2083 | -0.1250 | 0.3021 |  | *0.0938* | ***0.7083*** | *-0.0417* | *0.0000* | ***0.5000*** |
| **Org. BBCH 85** | 0.1042 | 0.0313 | -0.0208 | -0.0625 |  | ***0.8854*** | *-0.0833* | *-0.0729* | ***0.5521*** |
| **Org. BBCH 89** | **0.9063** | 0.4792 | -0.1563 | 0.4167 | 0.1250 |  | ***0.8958*** | ***0.9688*** | ***0.9479*** |
| **Biodyn. BBCH 81** | -0.0208 | -0.0521 | 0.4479 | -0.1667 | -0.1458 | **0.6563** |  | *-0.1563* | ***0.6771*** |
| **Biodyn. BBCH 85** | -0.0156 | -0.0521 | 0.0938 | 0.0313 | -0.2083 | 0.3229 | -0.1563 |  | ***0.8125*** |
| **Biodyn. BBCH 89** | **0.8542** | 0.3958 | -0.1563 | 0.4583 | 0.1875 | -0.0417 | **0.6354** | 0.3021 |  |

^a^Plots with clearly different (R > 0.5) or separated communities (R > 0.75) are printed in bold.
