# Supplementary material for: Are Epiphytic Microbial Communities in the Carposphere of Ripening Grape Clusters (Vitis vinifera L.) Different between Conventional, Organic, and Biodynamic Grapes?
Source: PLoS One. 2016 Aug 8;11(8):e0160852. doi: 10.1371/journal.pone.0160852 (PMC4976965; doi:10.1371/journal.pone.0160852)
Supplement: S8 Table — R values obtained for 2010 samples are shown below the diagonal, R values for 2011 samples are printed in italics above the diagonal. (DOCX) [file pone.0160852.s012.docx]

**S8 Table. Computation of R values after analysis of similarities between bacterial samples obtained from conventional, organic and biodynamic grapes sampled at three different stages of berry maturation.** R values obtained for 2010 samples are shown below the diagonal, R values for 2011 samples are printed in italics above the diagonal.^a^

|  | **Conven. BBCH 81** | **Conven. BBCH 85** | **Conven. BBCH 89** | **Org. BBCH 81** | **Org. BBCH 85** | **Org. BBCH 89** | **Biodyn. BBCH 81** | **Biodyn. BBCH 85** | **Biodyn. BBCH 89** |
| --- | --- | --- | --- | --- | --- | --- | --- | --- | --- |
| **Conven. BBCH 81** |  | *0.4375* | ***0.8333*** | ***0.7604*** | ***1.0000*** | *0.3542* | ***0.8646*** | ***0.5208*** | ***0.5000*** |
| **Conven. BBCH 85** | 0.2396 |  | ***0.5208*** | ***0.5313*** | ***1.0000*** | *0.2188* | ***0.7292*** | *0.4271* | *0.4063* |
| **Conven. BBCH 89** | **0.6042** | **0.5313** |  | ***0.9167*** | ***0.9286*** | *0.0000* | ***0.9063*** | ***0.8125*** | *-0.0208* |
| **Org. BBCH 81** | -0.0417 | 0.2917 | 0.4896 |  | *0.1786* | *0.4375* | *-0.0938* | *-0.1250* | ***0.5938*** |
| **Org. BBCH 85** | 0.3854 | 0.2813 | 0.3646 | 0.2500 |  | *0.3214* | *-0.0357* | *-0.0357* | *0.3929* |
| **Org. BBCH 89** | **0.6458** | 0.4167 | 0.2917 | 0.3958 | -0.0417 |  | ***0.5000*** | *0.2917* | *0.0000* |
| **Biodyn. BBCH 81** | 0.0938 | 0.4479 | **0.5625** | -0.0417 | 0.4583 | 0.4792 |  | *-0.1667* | ***0.5938*** |
| **Biodyn. BBCH 85** | 0.4271 | 0.3229 | 0.2292 | 0.1146 | 0.2083 | 0.2292 | -0.0521 |  | *0.4167* |
| **Biodyn. BBCH 89** | **0.6875** | 0.4583 | 0.4583 | **0.5729** | 0.2500 | 0.0208 | **0.5521** | 0.2813 |  |

^a^Plots with clearly different (R > 0.5) or separated communities (R > 0.75) are printed in bold.
